# Anatomical Exploration of the KI1 Acupoint: Implications for Medial and Lateral Plantar Nerve Stimulation

**DOI:** 10.3390/medicina60040535

**Published:** 2024-03-26

**Authors:** Sang Hyun Kim, Jin-Yong Joung, Young Ho Lee, Chang-Gue Son

**Affiliations:** 1 Catholic Institute for Applied Anatomy, The Catholic University of Korea, Seoul 06591, Republic of Korea; amalang@catholic.ac.kr; 2 Department of Korean Medicine, Korean Medical College, Daejeon University, 62, Daehak-ro, Dong-gu, Daejeon 34530, Republic of Korea; bluetxt1@gmail.com; 3 Department of Anatomy, College of Medicine, Chungnam National University, 266, Munhwa-ro, Jung-gu, Daejeon 35015, Republic of Korea

**Keywords:** KI1, acupuncture, medial plantar nerve, lateral plantar nerve, anatomy

## Abstract

*Background and Objectives:* This study aims to identify the precise anatomical location and therapeutic mechanisms of the KI1 acupoint (Yongquan) in relation to foot muscles and nerves, known for treating neurological disorders and pain. *Materials and Methods:* Dissection of six cadavers at Chungnam National University College of Medicine examined KI1’s relation to the foot’s four-layer structure. *Results:* The KI1 acupoint was located in the superficial and deep layers of the plantar foot, adjacent to significant nerves like the medial and lateral plantar nerves. Differences in the acupoint’s exact location between genders were noted, reflecting variances in foot morphology. KI1 acupuncture was found to stimulate the muscle spindles and nerve fibers essential for balance and bipedal locomotion. This stimulation may enhance sensory feedback, potentially improving cognitive functions and balance control. *Conclusions:* This anatomical insight into KI1 acupuncture underpins its potential in neurological therapies and pain management.

## 1. Introduction

Acupuncture is acknowledged globally as an effective complementary and alternative therapy, particularly beneficial for neurological and musculoskeletal conditions [1]. This traditional practice, now under the lens of modern scientific research, aims to blend ancient healing techniques with the latest medical knowledge. Anatomy plays a key role in this research, offering insights into how the human body works and how acupuncture can be effective [2].

Numerous studies on the mechanisms of acupuncture’s action and effects have sparked debate about the precise targets of acupuncture stimulation. Some studies suggest that acupoints are intricately linked to the nervous system [3], whereas other research highlights their critical role within muscle tissues for therapeutic effects [4]. One study reported over 90% of the 360 traditional acupuncture points correspond to myofascial trigger points, spots in muscle that cause pain and other symptoms upon compression [5].

Recent experimental findings have shed light on this debate, revealing that the pain-control effect of the widely utilized ST36 (Zusanli) acupoint in clinical settings is induced by the activation of specific sensory neurons that drive the vagal–adrenal axis [6]. For example, a study reported that HT7 (Shenmen) inhibition of cocaine-induced locomotion requires activation of A-fibers in the ulnar nerve [7]. Nevertheless, elucidating the workings of acupuncture/acupoints solely through the lens of nerves and muscles proves challenging, prompting the introduction of hypotheses based on quantum principles [8]. Concurrently, advancements in anatomical research have suggested the intriguing possibility that acupoints may correspond to neurovascular bundles [9]. Notably, a recent study revealed that acupuncture points can dynamically change in response to physiological and pathological states of the body [10].

**Figure 1:**
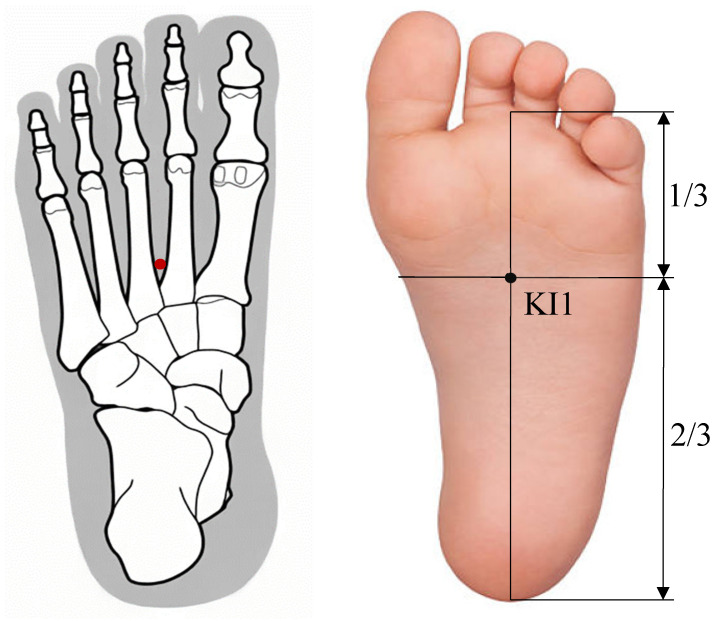
Location of the KI1 point (Yongquan). Located on the sole, the point is in a depression when the foot undergoes plantar flexion and in the anterior depression when the foot is flexed, situated approximately at the junction of the anterior one-third and the posterior two-thirds of the sole.

On the other hand, based on clinical and preclinical studies, the KI1 acupoint (Yongquan) is thought to be effective in improving consciousness disorders, enhancing neuronal synaptic plasticity, mitigating neuroinflammation, and reducing thermal hyperalgesia [11,12,13,14,15]. The location of the KI1 acupoint is approximately at the junction of the anterior third and the posterior two-thirds of the line connecting the heel to the web margin, between the 2nd and 3rd metatarsal bones [16] (Figure 1). However, anatomical studies, especially regarding the nerves and muscles as the basis for the therapeutic action of the KI1 acupoint, have been rare. This research aims to uncover the therapeutic mechanisms of the KI1 acupoint through precise anatomical studies on cadavers.

## 2. Materials and Methods

To elucidate the anatomical features of the KI1, ten cadavers (5 males and 5 females), donated to Chungnam National University College of Medicine for education and research, were preliminarily prepared. Among these, six cadavers (3 males and 3 females) without foot deformities such as hallux valgus (bunions) and severe muscular dystrophy in the foot were selected for dissection. The mean age of the cadavers was 83.3 for males and 85.7 for females. This study received approval from the Institutional Review Board of Chungnam National University (202106-BR-090-01). A study involving human cadavers was conducted following the acquisition of written informed consent from the deceased subjects’ next of kin. Since the sole of the foot has a structure of four layers [9], the structures related to KI1 were observed in each layer.

As part of our dissection process, we initially inserted a long needle at the KI1 acupoint, confirming that the needle emerged at the point one-third the distance from the plantar surface of the 2nd toe to the heel, between the 2nd and 3rd metatarsal bones on the dorsum of the foot. Additionally, the dissection aimed to precisely document the acupoint’s relation to surrounding structures, vital for understanding its therapeutic potential.

## 3. Results

**Figure 2:**
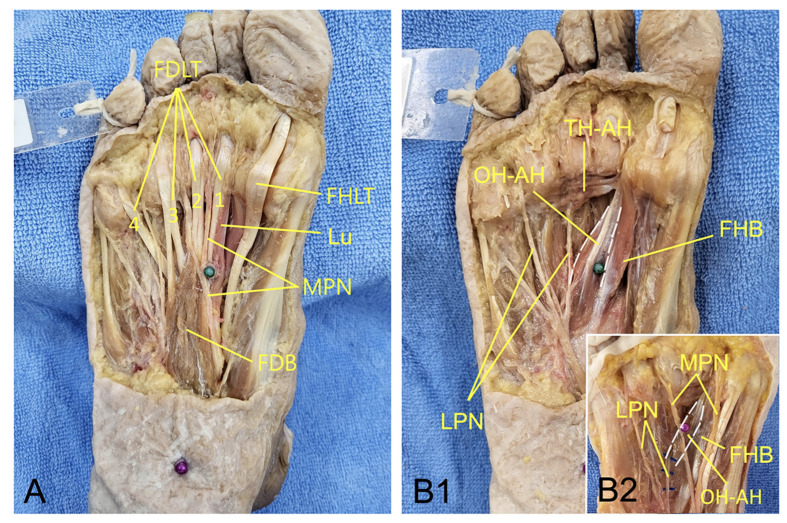
(**A**) Anatomical feature around the KI1 acupoint in the superficial (1st and 2nd) layers of the plantar foot, and in the deep (3rd) layer of the plantar foot in male (**B1**) and female (**B2**). (The FDB, FDLT, and FHLT were cut). FDLT, flexor digitorum longus tendon; 1, 1st FDLT; 2, 2nd FDLT; 3, 3rd FDLT; 4, 4th FDLT; FHLT, flexor hallucis longus tendon; Lu, lumbricalis; MPN, medial plantar nerve; FDB, flexor digitorum brevis; OH-AH, oblique head of adductor hallucis; TH-AH, transverse head of adductor hallucis; LPN, lateral plantar nerve; FHB, flexor hallucis brevis; dotted white line, medial and lateral borders of OH-AH; green pin head in A and B1, KI1 acupoint; purple pin head in B2, KI1 acupoint.

In the superficial (1st and 2nd) layers of the plantar foot, KI1 is located at the distal part of the belly of the flexor digitorum brevis, beside the lumbricalis, and at the proximal point between the 1st and 2nd tendons of the flexor digitorum longus. The medial plantar nerve passes near KI1 [Figure 2A]. In the deep (3rd) layer of the plantar foot, KI1 is located on the medial (great toe) side in males and on the lateral (little toe) side in females of the oblique head of the adductor hallucis [Figure 2B1,B2]. We also found that the lateral plantar nerve enters the oblique head of the adductor hallucis [Figure 2B1,B2]. These anatomical findings support that KI1 acupuncture can directly stimulate the medial plantar nerve, as well as the deep fasciae of these muscles innervated by the plantar nerve [17]. KI1 acupuncture can also stimulate the lateral plantar nerve that innervates the adductor hallucis in the 3rd layer of the plantar foot [18] [Figure 2B1,B2]. However, moxibustion at the KI1 acupoint may primarily stimulate the medial plantar nerve located in the superficial layers of the plantar foot.

The origins of the oblique head of the adductor hallucis are the medial process of the calcaneal tuberosity, the flexor retinaculum, and the plantar aponeurosis, while its insertion is at the base of the proximal phalanx of the great toe [17]. Interestingly, KI1 is located on the lateral side of the oblique head of the adductor hallucis in females, and on the medial side in males. This finding may reflect sex differences in foot shape, such as the broader width of the foot in males compared to females [19].

## 4. Discussion

This study highlights the neural and muscular structures targeted by acupuncture at the KI1 acupoint, revealing differences between male and female anatomies regarding this stimulation. Recent studies have shown that sensory nerves and connective tissues in the soles, crucial for detecting pressure and vibration, have a direct impact on the central nervous system [20]. This indicates that stimulating plantar nerves can notably improve motor and cognitive functions, presenting significant advantages for therapeutic rehabilitation [21].

Targeted stimulation at the KI1 acupoint not only activates local muscle spindles but also engages a wider neural circuitry, encompassing C and Aδ nerve fibers within the connective tissue, deep fascia, tendons, and muscles [17]. KI1 acupuncture directly excites muscle spindles, especially within the oblique head of the adductor hallucis, as well as C and Aδ fibers situated externally to the plantar muscles [22]. This activation is pivotal for maintaining equilibrium and supporting bipedal movement by relaying essential sensory data from the feet to the brain, thus initiating cognitive feedback [23].

Engaging the KI1 acupoint may amplify this sensory feedback, facilitating improved communication between the afferent nerves and the brain. This enhancement has the potential to refine balance control and augment cognitive abilities. A clinical study utilizing functional magnetic resonance imaging (fMRI) has discovered that foot stimulation not only enhances cognitive ability in elderly individuals [23], but also improves brain connectivity in individuals with Parkinson’s disease [16], thereby emphasizing the significance of this sensory input.

While the precise mechanisms through which the stimulation of C and Aδ fibers (innervated by plantar nerves) enhances brain functionality are yet to be fully understood, theories involving neuroplasticity and the adjustment of neurotransmitter or glucocorticoid levels have been suggested [24]. Recent research highlights that C fibers, rather than Aδ fibers, play a crucial role in acupoint sensitization, contributing to analgesia, cardiovascular regulation, and improving cognitive function via the dorsal root ganglia [25].

The concept of neuroplasticity, the brain’s ability to reorganize itself by forming new neural connections throughout life, offers a promising framework for understanding how acupuncture can produce lasting effects on brain function and structure [26]. Acupuncture’s ability to modulate neuroplastic changes may explain its efficacy in treating neurological conditions and enhancing cognitive abilities. Numerous studies utilizing MRI have revealed that acupuncture treatments initiate neuroplastic changes in brain-damaged areas and facilitate improvements in symptoms related to pain modulation and cognitive functions, indicating that repeated stimulation could result in prolonged enhancement of brain functionality and neuroplasticity [27].

Research is increasingly focusing on acupuncture’s effects on the central nervous system (CNS), extending beyond the local impacts at the site of needle insertion to include its influence on neurotransmitter modulation and the activation of the brain’s pain and reward systems. Recent studies have elucidated a neuroanatomical basis for the specific effects of electroacupuncture, particularly highlighting how stimulation at certain acupoints like ST36 can drive the vagal–adrenal axis, offering a targeted approach to modulate systemic inflammatory responses and potentially treat a variety of diseases [6]. The research provides critical insights into how electroacupuncture stimulation at the ST36 acupoint, but not at other locations like the abdominal ST25 acupoint, can specifically activate the vagal–adrenal anti-inflammatory axis in mice. This specificity is underpinned by the unique innervation patterns of PROKR2-Cre-marked sensory neurons that target the deep hindlimb fascia, such as the periosteum, which are crucial for activating hindbrain vagal efferent neurons and driving catecholamine release from the adrenal glands, thereby suppressing systemic inflammation induced by bacterial endotoxins.

Considering the variation in the KI1 acupoint location between males and females, which influences the specific muscles and nerves stimulated, it is reasonable to speculate that acupuncture’s therapeutic effects may differ by gender [28]. This speculation is supported by an fMRI study that showed gender-based differences in brain activation following acupuncture at knee acupoints, highlighting the role of anatomical and physiological distinctions in modulating acupuncture’s impact on neural pathways and muscle responses. However, the scope of this anatomically focused study does not extend to definitively explaining the gender-specific effects of the KI1 acupoint. Thus, further investigation is necessary to understand these gender-related differences in acupuncture’s therapeutic efficacy.

This study has several limitations. The reliance on anatomical dissections underscores the need for further in vivo research and studies involving living human subjects to accurately assess the physiological effects of acupuncture. The dynamic properties inherent in living tissue, crucial for directly observing the impacts on neural activities and pain management, cannot be fully replicated in cadaveric samples. Additionally, the study’s use of a limited number of cadavers, specifically three men and three women of advanced age, introduces potential biases. All participants were over 80 years old, which may not represent the broader population due to age-related physical conditions. There is a report showing foot-shape changes with age, especially in women [29]. Moreover, recent studies have observed variations in the structures of the sole in some anatomical cases, including the flexor digitorum accessorius muscle [30], which could alter the relationship between acupoints and their surrounding anatomy. These limitations restrict the generalizability of our findings and highlights the importance of considering a more diverse demographic and anatomical variations in future research.

## 5. Conclusions

In summary, the present study provides an anatomical exploration, focusing on the stimulation of both the medial and lateral plantar nerves, along with their innervating afferent fibers including C and Aδ. This research offers insights into how the KI1 acupoint may influence various physiological and neurological functions, thereby contributing to its therapeutic potential in clinical applications.

## Data Availability

Data available on request from the authors.
